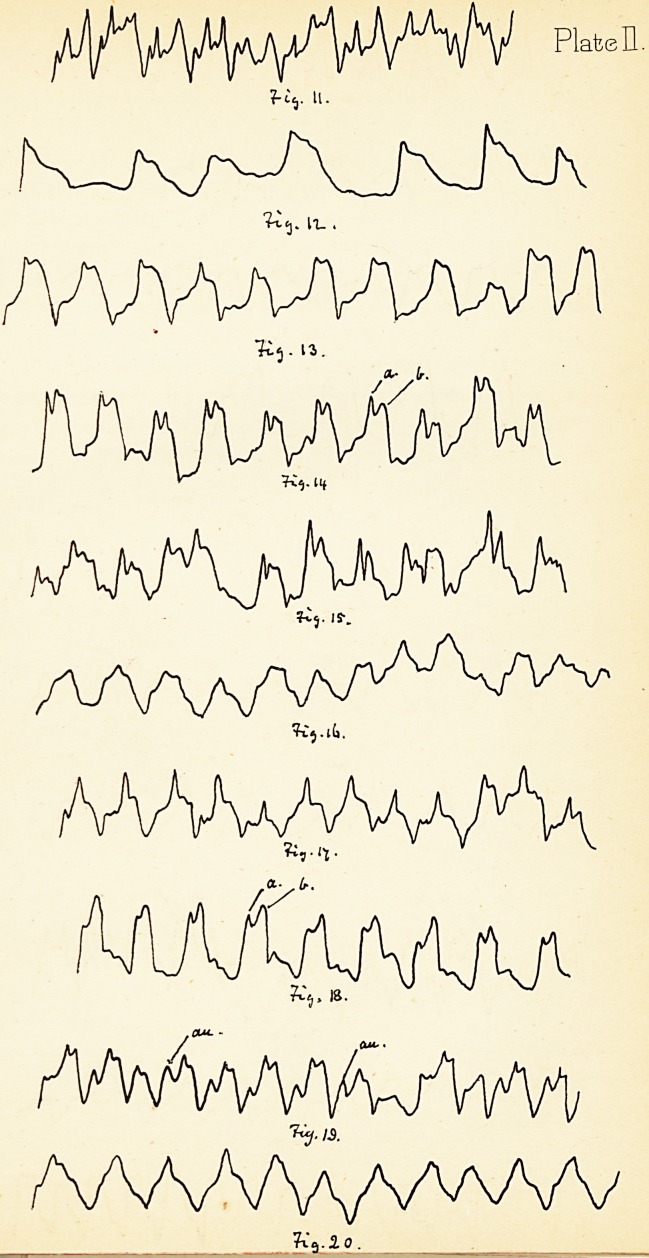# The Cardiograph in Medicine

**Published:** 1883-07

**Authors:** G. Munro Smith

**Affiliations:** Physiological Demonstrator at the Bristol Medical School


### THE CARDIOGRAPH IN MEDICINE.

BY

G. MUNRO SMITH, L.R.C.P. Lond., M.R.C.S.,

*Physiological Demonstrator at the Bristol Medical School.*

---

THE cardiograph is often spoken of as a "scientific toy," whose proper place is the physiological laboratory, where its tubes, levers, and bright brass cylinder and clock work add considerably to the appearance of the room, and help to amuse restless medical students and demonstrators. This opinion, however, is merely the result of ignorance; for a very slight knowledge of modern physiology will show what an important part this instrument has taken in elucidating the mechanism of the circulation, in correcting mistaken ideas, and in verifying and making accurate what was before loose conjecture. It has been wisely said that "the physiology of to-day is the pathology of to-morrow," and this is especially true in the domain of heart disease, where any additional knowledge of the action of the valves or of the auricular and ventricular contraction is certain before long to be turned to account therapeutically. Physiology is the parent of the pathology and treatment of heart disease, and any apparatus that can correctly interpret and make manifest the changing phases of the circulatory system must, *ipso facto*, be of use in medicine.

The tracings copied in this paper were chiefly taken at the Bristol Royal Infirmary. They were obtained, as much as possible, under similar conditions and with the same instrument. In many cases the results were unsatisfactory, and it must be owned that the cardiograph, unaided by the stethoscope, would be a dangerous guide; but without unduly pushing its claims I think we may safely say (1) that it verifies diagnosis gained by other means, in a striking manner, (2) that it often assists the diagnosis in doubtful cases, and (3) that it may be used as an index of treatment by showing the amount of regurgitation into the auricles and ventricles in mitral and aortic incompetence, the extent of hypertrophy of the walls of the heart, and the state of the general arterial system, as to tension, &c.

Space will not allow of a detailed description of the cardiograph, but it may be said that it consists essentially of two air-tight metal boxes, or drums, connected together by a flexible tube. Each drum has one of its sides made of stretched india-rubber, and, since the whole forms a continuous cavity containing air, if one of the drums be pressed by its elastic side against the apex-beat on the chest wall, the slightest movement of the heart will compress the air in the apparatus and cause a bulging of the india-rubber membrane on the second drum, and these movements are, in their turn, amplified by a light lever, and recorded on blackened paper which is made to travel at a uniform rate. The greatest precaution must be taken to apply the instrument exactly over the heart beat, otherwise a very different tracing or "cardiogram" will be obtained, for the chest walls are actually drawn in in parts of the area over the contracting venticle, at least in thin subjects. The chief drawbacks to the cardiograph are these: no two instruments give exactly the same results; that is to say, there is generally some difference in the height of the "up-stroke," the rapidity of the "down-stroke," &c., due to variations in the elasticity of the drum membranes, the length of the lever, the rate of motion of the recording cylinder, &c. Again, the instrument is somewhat bulky, requires practice and time, and is, moreover, sometimes alarming to the patient. A little trouble will overcome these difficulties. The subject may be thus divided:—(1) Analysis of normal tracings, (2) those taken from cases of mitral disease, (3) ditto from aortic, and (4) arterial tracings.

### ANALYSIS OF THE NORMAL HEART TRACING.

Tracings taken from apparently healthy people differ from each other considerably, according to the tranquillity of the circulation, the shape of the chest wall, the amount of fat under the skin, the time of the day and the state of the subject as regards rest, &c. In most cases, for instance, the tracing is very different after the drum has been kept for a minute or two against the chest wall. During the first 30 seconds or so, either from nervousness or involuntarily holding the breath, the mark made by the lever may show almost every variety of diminished tension and irregularity. Figs. 1, 2 and 3 were taken from a healthy student at the Medical School. The instrument was placed over the upex beat and kept there steadily during the whole time, and yet the three are as diverse as possible. No. 1 shows a pointed, irregular, jerky impulse. This is the result of the first few seconds of application. In No. 2, after the lapse of about 30 seconds, the curve is quieter, but still irregular; and No. 3 is a fairly normal tracing. The typical "normal" tracing should be taken from a subject accustomed to the machine. He should sit quietly, and should have been at rest for at least ten minutes previously. Muscular effort gives a double top to the tracing. Fig. 4 may be taken as a model for analysis. Each beat is represented by (1) the up-stroke, (2) the summit, and (3) the down-stroke. These are the three cardinal divisions of each tracing. The first (*a* to *b*) corresponds to the sudden "hardening" of the distended ventricle which is felt at the chest wall as the "beat." It may be looked upon as the commencement of the systole, but in reality it precedes the ventricular contraction. The ventricular wall, soft and flabby during diastole, suddenly becomes hard and tense and immediately afterwards contracts on the blood. The elevation (*au*), not always present, and very small in health, is due to the auricular contraction making itself felt in the ventricle. The second part, or summit (*b* to *c*), should be flat, not pointed, and might be conveniently described as the "plateau." It is rarely quite straight, and in disease may assume every kind of irregularity. This is the most important part of the tracing, and its history is worth careful study. The whole plateau corresponds to the ventricular contraction. During this period a struggle is going on between the ventricle on the one hand and the elastic force of the aorta on the other. The former is laboriously overcoming the resistance offered to it by the distended aorta. When the ventricle has nearly emptied itself it ceases from its effort, relaxes, and the lever falls. It will be seen in Fig. 4 that the first part of this tableland is slightly higher than the rest, partly because the contraction is at first more powerful, partly because the lever has been jerked up higher than it should have been by the impulse causing the up-stroke. Then there generally follow one or more irregularities due to the oscillations of the blood in the ventricle and arterial system, for it must be remembered that these now form one cavity, because the semilunar valves are widely open. The ventricle keeps up its contraction with very little diminution of force until the end of the plateau, when it withdraws from the struggle and the aorta is left the victor. The elastic force of the latter, which is very great, at once comes into play and the blood rushes back into the ventricle, but is almost immediately stopped by the semilunar valves. This obstacle sometimes gives a slight jerk to the heart, as seen in the 4th beat in Fig. 4. This is called the "aortic notch" or beat. The down-stroke (*c* to *d*, Fig. 4) represents the cessation of contraction and the relaxation of the heart. It may be rapid or gradual, straight or uneven, and each of these conditions has a special meaning. It is especially modified by the state of the valves.

### EFFECTS OF MITRAL DISEASE ON THE HEART TRACING.

I. *Regurgitation.*—When collecting tracings for purposes of analysis and comparison one soon realises how difficult it is to find a case of pure obstruction or regurgitation at any of the valvular orifices. The reasons for this are manifest; every influence which so modifies the valves as to impede the outward flow of blood is pretty sure to make them also incompetent; and conversely, the most common kinds of incompetence are associated with thickening of the valvular edges and therefore cause a certain amount of obstruction. For these reasons it may generally be assumed that whether a case be called obstruction or regurgitation it is not purely so. Figs. 5, 6, and 7 are examples of regurgitation with only a slight amount of obstruction, and the first two (Fig. 5 and 6)

are the most common type. The usual condition of things in this disease is as follows:—In the first place the left auricle and ventricle communicate more or less freely, and act, both in systole and diastole, as one cavity. In the second place, as the auricle has constantly to struggle with regurgitant blood, as well as with that which reaches it from the pulmonary veins, it becomes hypertrophied, and consequently its beat is more distinct than in health, and is made manifest in the apex tracing, as seen in (*au*) Fig. 5 and 6, and to a less degree in Fig. 7. I think this enlargement of the auricular notch is a fairly good index of the amount of backward flow into the auricle.

When the ventricle contracts, instead of having only one outlet for its volume of blood, it has two, the aorta and the auricle. In early stages of the affection this gives the ventricle less work to do, as, for example, when the mitral valves give way in aortic disease. But afterwards, when the lungs and the right side of the heart have become chronically distended, it has more to contend with than before, because it gets more blood sent into it. The result of having the auricle as an extra channel into which the blood can be driven is this: the struggle between the ventricular and aortic pressures is shortened; the ventricle with a short systole forces out its blood, partly into the auricle and pulmonary veins, partly into the aorta, and then, having emptied itself and fulfilled its duty, it retires from the contest; the aortic valves flap to with a jerk and the ventricle relaxes. Hence the second noticeable fact in the tracings, the absence of the plateau.

In consequence of the easier victory of the aortic tension the semilunar valves close with greater force and the second sound is consequently often sharper; at least in moderate regurgitation. This aortic jerk is shewn in the tracings (Fig. 5 and 7).

In some cases the apex is double, as seen in Fig. 8, which is in many ways peculiar. The tracings taken from the radial, carotid and subclavian arteries of this patient shewed a markedly square top, and the heart disease was probably secondary to general arterial thickening. The cardiogram indicates hypertrophy by the height of the upstroke; mitral regurgitation by the auricular notch (*a*) and the pointed apex (*b*), and feeble tension by the second elevation (*c*), for it is quite possible to have great obstruction and feeble tension. Fig. 9 is the tracing of the left subclavian of this patient, and is interesting when compared with Fig. 8.

Mitral regurgitation, therefore, seems to affect the heart tracing chiefly in three ways, (1) by giving an upstroke broken by the mark of the auricular contraction, (2) by absence or irregularity of the plateau, and (3) by the presence in many cases of a marked aortic notch.

When there is both marked obstruction and regurgitation the tracing is very different from the above, and resembles the normal type in many points. Fig. 10 is taken from a case of double mitral murmur, and might represent a healthy tracing were it not for the auricular notch and the signs of hypertrophy. The rhythm in the above is too regular for simple mitral regurgitation.

II. *Mitral Obstruction.*—As before stated, it is difficult to conceive such a condition as would lead to mitral obstruction with no concomitant regurgitation. Consequently we cannot expect to find a special tracing characteristic of the first alone. Even in those cases in which there is a bruit presystolic in rhythm, and no systolic one following, the cardiograph shows that regurgitation exists. In fact it seems doubtful if the so-called presystolic bruit has any existence. Herard and Constantin Paul argue that this bruit is not caused by the forcible contraction of a hypertrophied auricle, but by the early part of the ventricular contraction, preceding the time of greatest hardening of the ventricle, which would be felt as the apex beat. If this be true, the presystolic bruit would indicate regurgitation into the auricle. Fig. 11 is taken from a somewhat pronounced case of apparent presystolic murmur, followed by a systolic. The great irregularity of the plateau and ascent are to be noticed. There was a thrill in this case, felt over the auricular as well as ventricular region. The violent auricular effort imparts to the heart a general vibration. In this Fig. the two chief elevations are due to the low tension in the ventricle, and mean regurgitation. I think it can be laid down as a rule that if a murmur is heard most distinctly at the apex, and if the tracing obtained resemble Fig. 11, the diagnosis is rather that of regurgitation than obstruction, whether the murmur appear to precede the systole or not.

In consequence of the narrowed or obstructed auriculo-ventricular orifice the regurgitation is sometimes interfered with as well as the flow in the normal direction. There is consequently a plateau or rounded top occasionally present, as in Fig. 13. In Fig. 12 there is a small irregular top also in some of the beats, but the majority are pointed. According to Prof. Marey* the tracing ought to show a small upstroke and an absence of the auricular contraction notch. But, he adds, "Toutes ces suppositions sont à vérifier." †

* *Circulation du sang.*, chap. xlii. † *Ibid*, p. 693, op. cit.

### EFFECTS OF AORTIC VALVULAR DISEASE ON THE HEART TRACING.

1. *Regurgitation.*—These tracings are very various, partly because the amount of regurgitation modifies the cardiac cycle according to its amount, and partly because of the great frequency of secondary mitral derangement; what may be called the "safety-valve action" of the mitral.

In moderate cases two points may be noticed: the first is obvious, and is merely the result of hypertrophy, namely, increased height of the upstroke. The second is a double apex. This is well seen in Fig. 14. Its mechanism may be thus explained. When the ventricle has finished its contraction, which is marked by the "first apex" in the tracing, it begins to relax, and the lever begins to fall. Normally this descent is straight, or only interrupted by a slight aortic notch, because the closure of the semi-lunar valves prevents any filling of the ventricle; but when these valves allow the blood to flow back the ventricle is again partly distended and the lever again rises. It is the shock of the regurgitant blood against the ventricular walls that causes the second apex (*b* in Fig. 14). This is taken from a severe case, and the second beat is well marked. I think the size of this aortic elevation is a fairly good criterion of the amount of regurgitation, a thing that is not always easy to detect otherwise, and the prognosis and treatment of the case would both be modified by this knowledge. Fig. 15 is taken from the same patient as Fig. 14, but after the exertion of sitting up in bed and having the back examined. It is interesting to compare this with Fig. 1, and to notice the similarity. The latter was taken from a heart that was merely "nervous."

Another feature of these tracings, but only an occasional one, is great irregularity of the summit and downstroke. Finally mild cases of aortic regurgitation may give a square top, and almost normal upstroke and downstroke.

2. *Aortic obstruction.*—Tracings show generally a long sloping upstroke occasioned by the laborious contraction of the handicapped ventricle, broken by occasional oscillation of the arterial pressure against which it is contending (Fig. 16).

The plateau may be rounded, short or long. When no regurgitation exists it is probably straight, and fairly long. Fig. 17 is introduced as a somewhat common form of tracing, taken from a case of aortic constriction modified by mitral regurgitation. On the whole the cardiograph is unsatisfactory in this disease.

*Arterial tracings in heart disease.*—The following figures were taken from different arteries, chiefly the carotid, with the cardiograph. They show distinctly the nature of the cardiac lesion.

Fig. 18 from a case of double aortic and regurgitant mitral disease shews hypertrophy (as evinced by size of upstroke), and a peculiar plateau, the second part of which (marked *b*) is apparently the result of the great aortic regurgitation that existed.

Fig. 19 is from a case of mitral regurgitation. The points worthy of notice are:—(1) The irregularity of rhythm; (2) The marked auricular systole (*au*); and (3) The oscillation of the plateau. Some small "abortive" pulsations are seen.

Fig. 20 is taken from the subclavian artery of a man suffering from aortic obstruction and slight regurgitation. There was also a systolic bruit at the apex, which

Fig. 1

Fig. 2.

Fig. 3.

Fig. 4.

Fig 5.

Fig. 6.

Fig. 7.

Fig. 8.

Fig. 9.

[illegible]

Fig. 11.

Fig. 12.

Fig. 13.

Fig. 14

Fig. 15.

Fig. 16.

Fig. 17.

Fig. 18.

Fig. 19.

Fig. 20.

accounts for the small notch in the upstroke. The gradual ascent, the rounded top, and the sloping downstroke are worthy of notice.

I have introduced these three last figures because they appeared to me to be unusual. The sphygmograph has already indicated most of the characteristics of abnormal arterial tracings.

Finally, my only apology for bringing together and endeavouring to explain the above cardiograms is that so very little has been done, especially in England, in what might become a fruitful field of clinical work.

## Figures and Tables

**Fig. 1 Fig. 2. Fig. 3. Fig. 4. Fig 5. Fig. 6. Fig. 7. Fig. 8. Fig. 9. Fig. 10. f1:**
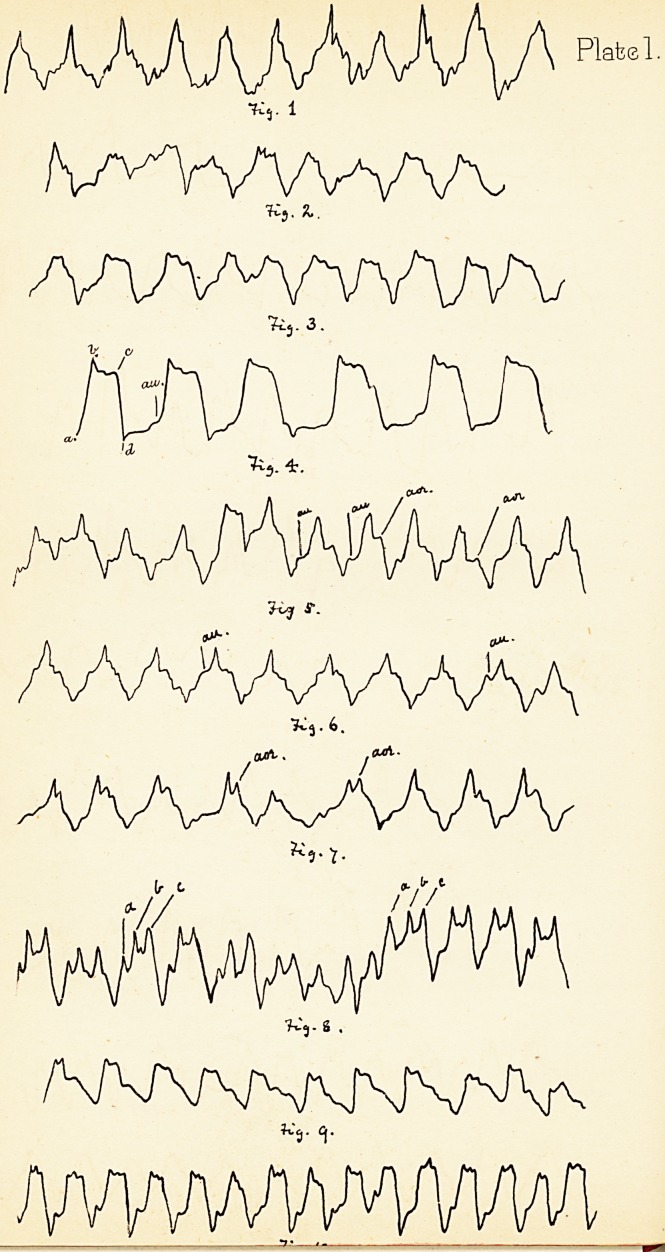


**Fig. 11. Fig. 12. Fig. 13. Fig. 14 Fig. 15. Fig. 16. Fig. 17. Fig. 18. Fig. 19. Fig. 20. f2:**